# Dysbiosis-Related Advanced Glycation Endproducts and Trimethylamine N-Oxide in Chronic Kidney Disease

**DOI:** 10.3390/toxins13050361

**Published:** 2021-05-19

**Authors:** Kensei Taguchi, Kei Fukami, Bertha C. Elias, Craig R. Brooks

**Affiliations:** 1 Division of Nephrology and Hypertension, Vanderbilt University Medical Center, Nashville, TN 37232, USA; bertha.elias@vumc.org (B.C.E.); craig.brooks@vumc.org (C.R.B.); 2 Division of Nephrology, Department of Medicine, Kurume University School of Medicine, Kurume 830-0011, Japan; fukami@med.kurume-u.ac.jp

**Keywords:** gut microbiota, dysbiosis, AGEs, RAGE, TMAO, chronic kidney disease

## Abstract

Chronic kidney disease (CKD) is a public health concern that affects approximately 10% of the global population. CKD is associated with poor outcomes due to high frequencies of comorbidities such as heart failure and cardiovascular disease. Uremic toxins are compounds that are usually filtered and excreted by the kidneys. With the decline of renal function, uremic toxins are accumulated in the systemic circulation and tissues, which hastens the progression of CKD and concomitant comorbidities. Gut microbial dysbiosis, defined as an imbalance of the gut microbial community, is one of the comorbidities of CKD. Meanwhile, gut dysbiosis plays a pathological role in accelerating CKD progression through the production of further uremic toxins in the gastrointestinal tracts. Therefore, the gut-kidney axis has been attracting attention in recent years as a potential therapeutic target for stopping CKD. Trimethylamine N-oxide (TMAO) generated by gut microbiota is linked to the progression of cardiovascular disease and CKD. Also, advanced glycation endproducts (AGEs) not only promote CKD but also cause gut dysbiosis with disruption of the intestinal barrier. This review summarizes the underlying mechanism for how gut microbial dysbiosis promotes kidney injury and highlights the wide-ranging interventions to counter dysbiosis for CKD patients from the view of uremic toxins such as TMAO and AGEs.

## 1. Introduction

Chronic kidney disease (CKD) has emerged as a major public health concern that affects an estimated 37 million people in the United States [1,2]. Not only is CKD the 9th leading cause of death in the U.S., but it also contributes to several diseases such as heart disease [3], stroke [4], cancer [5], and cognitive impairment [6], thereby increasing mortality rates in patients with CKD [7]. Therefore, preventing the new onset of CKD or inhibiting the progression of CKD is of paramount importance for public health. It has been shown that inappropriate activation of the renin-angiotensin system, sympathetic nerve activation, and upregulated inflammatory cytokines are implicated in the progression of CKD [8,9,10]. In addition to the traditional risk factors for CKD progression, recent studies have revealed that gut microbial dysbiosis, defined as an “imbalance” in the gut microbial community, is also one of the key contributors to accelerating CKD progression [11].

Substances that are typically excreted or metabolized by the kidney accumulate as renal function declines. If the substances induce clinical symptoms of uremia, they are referred as to “uremic toxins.” Advanced glycation endproducts (AGEs), a heterogeneous group of molecules formed by a non-enzymatic reaction between reducing sugars and amino acids, lipids, and DNA, are considered as one of the representative uremic toxins [12]. AGEs accumulate in visceral organs crosslinking with matrix molecules and disrupting matrix-matrix and matrix-cell interactions, resulting in organ dysfunction in CKD [13]. Also, through the interaction with the receptor for AGEs (RAGE), AGEs promote further oxidative stress and stimulate several intracellular signaling molecules, leading to the production of inflammatory and profibrotic cytokines [14]. Another uremic toxin, trimethylamine N-oxide (TMAO), which is a degradation product of choline and L-carnitine, is shown to accumulate when renal function declines and correlates with enhanced cardiovascular mortality in CKD patients [15,16]. An elevated TMAO value is not only a predictor of cardiovascular disease, but it also promotes renal fibrosis, the main feature of CKD [17].

The human gut microbiota consists of approximately 1000 species and there are ~100 trillion bacteria in the gut, the genetic information of which is 150 times larger than that of the host [18]. With better understanding as technology advances, gut microbiota seems to not only maintain homeostasis, but also to be involved in the pathology of several human diseases including obesity [19], hypertension [20], cancer [21], depression [22], and cardiovascular diseases [23]. Further, recent studies have shown that gut microbiota dysbiosis including loss of beneficial microbes, expansion of pathobionts, and loss of microbiome diversity, is one of the common features in patients with CKD, possibly due to metabolic acidosis, accumulation of uremic substances in the intestine, volume overload-induced edema of intestinal epithelial cells, and frequent use of antibiotics and oral iron agents [24]. It has been found that the proportions of the bacteria possessing urease, uricase, and p-cresol- and indole-producing enzymes are increased in CKD, leading to uremic toxicity and systemic inflammation [25]. Many substances secreted by the microbiota become uremic toxins in the setting of CKD and can be absorbed into the body [26,27]. Thus, gut microbiota impairment plays a pathological role in the development of uremia in CKD patients and represents a potential therapeutic target for CKD progression.

A recent study has demonstrated that an AGEs-rich diet affects the gut microbiota [28], which may be linked to TMAO production [15]. Further, accumulation of AGEs was found in the gastrointestinal tract of CKD patients in association with gut microbiota dysbiosis, possibly leading to further progression of CKD [29]. Understanding the mutual relationship between gut microbiota dysbiosis and uremic toxins such as AGEs and TMAO is of great importance for preventing kidney injury and developing new therapeutic options for CKD patients. This review highlights the underlying mechanisms by which the reciprocal regulation of gut microbiota dysbiosis and uremic toxins drives CKD progression.

## 2. Gut Microbiota, AGEs, TMAO, and Inflammation

### 2.1. Dysbiosis in CKD

A number of studies have shown that CKD affects the distribution and makeup of gut microbiota, which is linked to dysbiosis [30]. Although the results of clinical studies may vary depending on the ethnicity, medical treatment, and lifestyle, it is generally accepted that bacterial species that produce urease (*Proteobacteria, Clostridiaceae,* and *Actinomycetales*), uricase (*Actinomycetales* and *Proteobacteria*), and indole or p-cresol forming enzymes (*Clostridiaceae* and *Prevotellaceae*) thrive, whereas species containing butyrate-forming enzymes (*Roseburia*, *Faecalibacterium*, *Clostridium*, and *Coprococcus*) are reduced in CKD [25]. Urease-producing bacteria including *Clostridiaceae* are known to hydrolyze urea in the gut to form ammonia, which, in turn, is converted to ammonium hydroxide. The high burdens of ammonia and ammonium hydroxide in the gut are associated with the alteration of gut microbiota and disruption of the intestinal epithelial tight junction [11]. Besides this, indole- or p-cresol-forming enzymes are responsible for metabolizing tryptophan and tyrosine into indole and p-cresol, respectively, in the gut. Then, in the liver, indole and p-cresol are converted to indoxyl sulfate (IS) and p-cresol sulfate (PCS), well-documented uremic toxins [31]. Uremic toxins are substances that are usually filtered and excreted by the kidneys, whereas as glomerular filtration rate (GFR) declines in CKD, these compounds accumulate and exert their deleterious effects on various organs. In fact, the serum concentration of IS and PCS are positively correlated with serum creatinine, a marker of GFR, and the patients with a later stage of CKD showed a higher concentration of IS or PCS. Thus, in addition to the loss of the ability to remove uremic toxins due to decreased GFR, the expansion of such bacterial species producing uremic toxins in the gut of CKD is linked to further accumulation of uremic toxins in the body.

The possible mechanisms for how the gut microbiota change in CKD are as follows: (1) accumulated uremic toxins perturb the composition of gut microbiota [11,32]; (2) metabolic acidosis; (3) therapeutic agents such as oral iron supplements [33], chelating agents [34], antibiotics [35], and proton-pump inhibitors [36]; (4) reduced bowel movement due to CKD–induced edema and ischemia; (5) lack of dietary fiber intake [37]; (6) prolonged intestinal transit time [24]. A recent investigation using microbiome analysis showed that the proportions of *Eggerthella lenta* and *Fusobacterium nucleatum* were increased in patients with end-stage renal disease (ESRD) when compared with healthy individuals, and fecal transplantation with the two species into rats with CKD exacerbated renal fibrosis and increased the production of uremic toxins [38], supporting the gut-kidney axis theory. Several systematic reviews and meta-analyses have also demonstrated that probiotic, prebiotic, and synbiotic supplements in the CKD population improve a circulating inflammation marker (C-reactive protein), oxidative stress markers (malondialdehyde, glutathione, and total antioxidant capacity), and lipids profiles [39,40]. Indeed, Rossi et al. conducted a randomized, double-blind trial to investigate the efficacy of synbiotic interventions in patients with CKD stage 4–5, and determined that with synbiotic supplementation, an enrichment of *Bifidobacterium* and depletion of *Ruminococcaceae* were observed with a decrease in the serum concentrations of IS and PCS [41].

CKD-induced dysbiosis is thought to accelerate the progression of CKD to ESRD through the production of uremic toxins, giving rise to a vicious cycle between CKD and dysbiosis. Once ESRD is established, uremic toxins still remain a major problem and source of uremic symptoms as they are not cleared by blood purification therapies, such as dialysis. Although an infusion of ibuprofen [42] or reduction of blood pH [43] are shown to increase the clearance of protein-bound uremic toxins during single dialysis treatment, no specific therapeutic option to enhance the removal of protein-bound uremic toxins has yet been established [44]. Thus, creating new interventions targeting the production process of uremic toxins is promising and needs to be further investigated.

### 2.2. Disruption of Gut Epithelial Barrier in CKD

**Figure 1:**
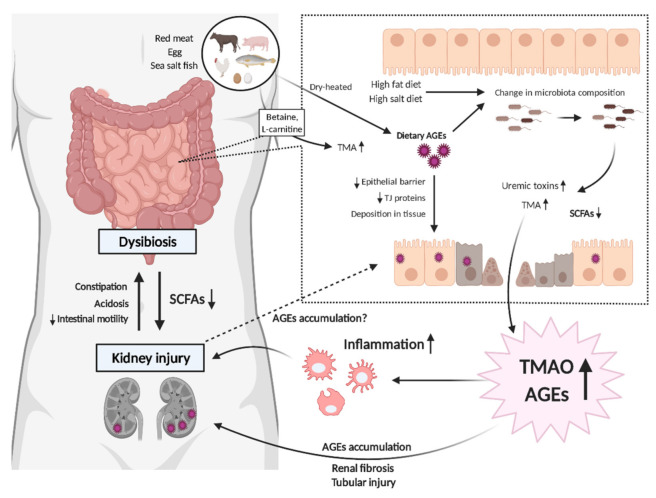
Schematic of the mutual link between gut dysbiosis and uremic toxins in chronic kidney disease (CKD). Excess dietary intake of advanced glycation endproducts (AGEs) affects the composition of the gut microbiome, leading to further uremic toxin production, which results in kidney injury. AGEs deposition in the gastrointestinal tract also disrupts the epithelial barrier, allowing bacterial components and endotoxins to flow into the systemic circulation, which, in turn, leads to other organ damage. The high burdens of uremic toxins such as trimethylamine N-oxide (TMAO) or AGEs are linked to progressive tubular injury and renal fibrosis, which are associated with the development of CKD to end stage renal disease.

Disruption of the gut epithelial barrier, referred to as a “leaky gut”, is another intestinal complication related to CKD [45]. The buildup of uremic toxins is thought to weaken the intestinal epithelial barrier, allowing bacterial components derived from intestinal bacteria, such as DNA, lipopolysaccharide (LPS), and endotoxins, to flow into systemic circulation through the leaky gut. IS induces systemic inflammation, leading to cellular injury in vascular endothelial cells, and promotes arteriosclerosis with an increase in thrombus formation (Figure 1) [46]. Kikuchi et al. demonstrated that a high serum concentration of phenyl sulfate, a protein-bound uremic solute, was associated with the progression of albuminuria in 362 patients with diabetic kidney disease (DKD) [47]. Besides endothelial dysfunction, IS induces cardiac fibrosis, thus affecting cardiac function [48]. AST-120, an oral charcoal adsorbent, was shown to attenuate cardiac hypertrophy and cardiac fibrosis by inhibiting the absorbance of uremic toxins in a rodent CKD model [49]. A meta-analysis concluded that serum IS and PCS levels strongly correlate with overall mortality in CKD patients [50]. The serum concentration of phenylacetylglutamine, a recently identified colonic microbial metabolite from amino acid fermentation, also predicts the prevalence of cardiovascular diseases [51]. These findings provide a link between the multiple organ damage seen in CKD patients and gut microbiota-derived uremic toxins.

As CKD progresses, endotoxins such as LPS flow into the body and function as pathogen-associated molecular patterns (PAMPs) [52]. A number of clinical investigations have demonstrated that individuals with a high concentration of serum endotoxins are more likely to develop cardiovascular diseases (CVD) [53]. Also, PAMPs and damage-associated molecular patterns (DAMPs) are involved in the progression of several types of kidney diseases including DKD through interaction with pattern recognition receptors such as RAGE and the toll-like receptor (TLR) [54]. In fact, injection with LPS worsened kidney function in streptozotocin-induced diabetic mice, and upregulation of the vascular endothelial growth factor (VEGF) in the PTECs of a diabetic kidney was further enhanced by the administration of LPS [55]. Recently, Nakano et al. revealed interesting findings, using intravital imaging by 2-photon microscopy, that LPS disrupts the tight junction of PTECs and induces paracellular leakage of filtered molecules and interstitial accumulation of extracellular fluid, leading to oliguria [56]. Interestingly, tubule-specific deletion of TLR-4, an LPS-binding receptor, retained the efficacy of intravenous fluid treatment in LPS-induced acute kidney injury (AKI) [56]. Although it is still not clear whether this theory is applicable to intestinal epithelial cells, the idea could provide an explanation for the prevalence of the “leaky gut” in CKD.

CKD-related dysbiosis and intestinal barrier dysfunction contribute to the progression of CKD. Therefore, there are several ongoing clinical trials attempting to improve dysbiosis by using prebiotics or probiotics in CKD patients [41,57,58].

### 2.3. Gut Dysbiosis and Inflammation

There are many overlapping processes that drive the progression of CKD to ESRD such as renin-angiotensin system activation, sympathetic hyperactivity, mitochondrial dysfunction, and so on [8,9,10]. In addition to the traditional risk factors, subclinical inflammation can be considered one of the contributors to the progression of CKD; thus, this section focuses on the links between dysbiosis, uremic toxins, and inflammation in CKD. The subclinical inflammation in CKD is characterized by an increase in inflammatory markers such as cytokines, acute-phase proteins, and adhesion molecules [59]. Chronic subclinical inflammation in CKD can be the result of increased production and decreased elimination of proinflammatory cytokines [60], gut dysbiosis [45], reactive oxygen species (ROS), metabolic acidosis [61], and alteration of adipose tissue metabolism [62], or a combination of these factors. Besides an increase in uremic toxin-producing bacteria, a concomitant reduction in the species generating beneficial short-chain fatty acids (SCFAs) including butyrate is one of the characteristics of CKD-related dysbiosis [63,64]. SCFAs are saturated fatty acids with a chain length ranging from one to six carbon atoms, produced by fermentation of dietary fiber in the colon. Recent studies provide evidence for gut microbiota producing the SCFAs influencing the severity of AKI by regulating the immune and inflammatory responses [65]. Mishima et al. demonstrated that intestinal SCFA production was dramatically reduced in germ-free mice, which worsened adenine-induced kidney injury [66]. Meanwhile, supplementing with exogenous SCFAs not only reduced ROS, but also suppressed cytokines and chemokines such as interleukin (IL)-1β, IL-6, tumor necrosis factor (TNF)-α, and monocyte chemoattractant protein-1 (MCP-1) [66]. Intriguingly, in vitro experiments showed that SCFAs prevented dendric cells’ maturation with inhibition of CD4^+^ and CD8^+^ T cell proliferation [67], indicating that SCFAs seem to directly modulate immune cells’ function and regulate cytokine/chemokine production. In addition to CKD-related dysbiosis characterized by lack of SCFA-producing bacteria, dietary restriction of fermentable fiber such as potassium-rich fruits and vegetables in CKD leads to the depletion of the bacteria that convert indigestible carbohydrates to SCFAs [63,64]. The most recent investigations demonstrated that dietary intake of sulfur-containing amino acids inhibited the function of tryptophanase, a secreted enzyme that catalyzes the degradation of tryptophan to indole, pyruvate, and ammonia, which resulted in a reduction in uremic toxin production, leading to the prevention of tubular injury in an adenine-induced CKD model [68]. Similarly, a number of studies have shown the efficacy of interventions targeting dysbiosis for slowing down CKD progression. Dietary supplementation with resistant starch—which is a carbohydrate that resists digestion in the small intestine and ferments in the large intestine, acting as a prebiotic—ameliorated kidney injury in a type 2 diabetic rodent model [69] and a CKD rat model [70]. Lubiprostone, a chloride channel activator that can improve the symptoms of constipation, was also shown to prevent tubular injury and GFR decline with increases in proportions of *Lactobacillus* and *Prevotella* in an adenine-induced CKD model [71]. Thus, a clinical trial to examine the inhibitory efficacy of lubiprostone (UMIN000023850) is currently underway.

With respect to AKI, Jang et al. showed that structural injury and functional decline following ischemic reperfusion injury (IRI) were more severe in germ-free mice with enhanced CD8^+^ T cells trafficking into the injured kidney. Reconstituting microbiota by adding fecal material from control mice to the germ-free mice attenuated histological damage, which led the authors to conclude that regulatory T cells may not be stimulated or mature in germ-free mice during development, thus amplifying renal damage [72]. In contrast, Emal et al. demonstrated that treatment with broad-spectrum antibiotics like ampicillin, metronidazole, neomycin, and vancomycin reduced microbial diversity and protected mice against IRI-induced kidney injury. This was achieved by suppressing the maturation status of F4/80^+^ renal-resident macrophages, well-known immune cells involved in the detection, phagocytosis, and destruction of bacteria and other harmful organisms, as well as by reducing the release of chemokines. Fecal material transplantation from control mice to antibiotic-treated mice abolished this protective effect [73]. Although previous animal data depend on whether germ-free animals or broad-spectrum antibiotic treatment was utilized for the removal of gut microbiota, the gut microbiota seem to be associated with inflammation, leading to modulation of the outcome of kidney injury in AKI, as well.

### 2.4. AGEs–RAGE Axis and Inflammation

A non-enzymatic reaction between reducing sugars such as glucose and the α-amino group or lysine residue at the N-terminal of amino acids is an initial step in the synthesis of AGEs [74,75]. It is followed by the formation of a Schiff base and Amadori compounds, which in turn, undergo various reactions such as dehydration, condensation, oxidation, and reduction. AGEs are finally generated with intermolecular crosslinking in visceral organs. This was first reported by a French chemist, Louis-Camille Maillard, in 1912, and extensively studied in the area of food chemistry. However, AGEs have begun to receive greater attention after the discovery of hemoglobin A1C, a glycated hemoglobin and a marker for type 2 diabetes [76,77]. Since then, research studies have shown that proteins with a slow turnover rate in the body, like collagens exposed to reducing sugars for a long period, have lysine residues that are AGEs-modified, which induces protein polymerization, reduced solubility, and susceptibility to proteases. Thus, AGEs are rarely degraded, and crosslinking of AGEs leads to irreversible organ damage [78].

There are two main drivers of AGE generation, namely, glycation and oxidative stress. Pyrraline and crosslines, the major AGEs, are produced via a glycation-dependent pathway [79]. Thus, hyperglycemia in diabetes is closely associated with the production of AGEs. In recent years, oxidative stress has also been shown to promote the synthesis of AGEs. N(ε)-carboxymethyl lysine (CML), a well-established AGE, is generated after the oxidative cleavage of Amadori compounds or through the auto-oxidation of glucose [80]. In fact, CML accumulation has been observed in the glomeruli of DKD, as well as hypertensive nephropathy [81,82] and lupus nephritis, in humans and experimental rodents [83]. RAGE, the main receptor for AGEs, is a type I transmembrane receptor and is expressed on the surface of several kidney cells including PTECs, mesangial cells, podocytes, and endothelial cells [81,84,85,86]. In addition to the intrinsic kidney cells, RAGE is also expressed on the membrane of immune cells such as monocytes [87], macrophages [88], and T and B lymphocytes [89,90]. The interaction between RAGE and its ligands is shown to directly activate immune cells, causing inflammation and the development of autoimmune diseases [91]. Since AGEs are one of the ligands of RAGE, AGEs promote the secretion of proinflammatory cytokines, such as IL-1α, IL-6, and TNF-α, through the activation of NF-ĸB, leading to the amplification of systemic inflammation [92,93]. AGEs also delay spontaneous apoptosis of monocytes, thus, contributing to the development of inflammatory responses [94]. Considering that RAGE also recognizes a wide range of DAMPs [93], it is likely that DAMPs derived from gut microbiota bind to RAGE, inducing immune cells to migrate and proliferate. With respect to acute inflammation, the genetic deletion of RAGE attenuated the hepatic expression of pro-inflammatory cytokines with a better survival rate compared to wild-type mice in LPS/D-galN-induced acute liver injury [95]. The outcome of a clinical research study supported the concept that there is a positive correlation between the expression levels of RAGE and E-selectin, endothelin-1, and TNF-α in septic AKI patients [96]. In addition, RAGE was implicated in chronic inflammation. Durning et al. demonstrated that RAGE enhanced the inflammatory function of T cells, and that its increased levels in patients with type 1 diabetes may account for the chronic autoimmune response [97]. In another study, old RAGE knockout (KO) mice exhibited a decrease in the renal concentration of proinflammatory cytokines compared to old wild-type mice, and attenuated glomerulosclerosis, a marker of aging kidneys [98]. RAGE expressed on various cell types plays a central role in amplifying and prolonging inflammatory responses through engagement with AGEs (Figure 1).

RAGE is linked to the development of human autoimmune diseases such as systemic lupus erythematosus (SLE) [91] and arthritis [99]. Tian et al. demonstrated that HMGB-1 released from necrotic or dead cells in autoimmune diseases activates autoreactive B cells and plasmacytoid dendritic cells, and augments inflammatory cytokines secretion through TLR-9 and RAGE [100]. Clinical research also showed that the polymorphism of the RAGE gene is correlated with the severity of SLE and the progression of lupus nephritis [101]. In vitro data showed that peripheral blood mononuclear cells (PBMCs) incubated with serum from lupus nephritis patients enhance the secretion of INF-1α, which is reduced by RAGE-Fc or a monoclonal antibody to RAGE [100]. Thus, a number of studies provide clear evidence that RAGE is a key contributor to inflammation and can be a potential therapeutic target for regulating the massive inflammation in CKD.

### 2.5. TMAO and Inflammation

The dietary intake of TMAO precursors such as choline, betaine, and L-carnitine, is an important factor for TMAO production. Choline is higher in animal-derived food as compared to plant-based food on a per unit of weight basis, and specifically abundant in liver, eggs, beef, fish, pork, chicken, and milk [102]. Betaine is found in vegetables, animals, and microorganisms, and is a significant component in spinach, wheat, sugar beets, and shellfish [103,104]. L-carnitine is found in many animal products, but especially, red meat has high levels. Small amounts of L-carnitine are also found in chicken, milk and dairy products, fish, beans, and avocado. The L-carnitine content of red meat and fish is not affected by freezing or cooking [105]. These precursors are converted by gut microbiota into an intermediate compound known as trimethylamine (TMA) in the gastrointestinal tract, which is then absorbed and delivered to the liver. There, it undergoes oxidization by Flavin-dependent monooxygenase3 to generate TMAO [106], which is then transported to the brain, muscle, kidney, and intestine [107]. Since TMAO is excreted into the urine, the serum concentration of TMAO is known to be elevated as renal function declines [17], which correlates with an increased risk of cardiovascular disease in general, and especially in patients with CKD [108]. Thus, TMAO has been considered as a uremic toxin as well.

Perturbations of the intestinal microbiota composition in both human and experimental CKD have demonstrated a significant elevation of TMAO, which is linked to an increased burden of inflammation as well as renal disease progression [109]. Interestingly, high-salt and high-fat diets (HFDs), both of which are characteristics of the current Westernized diet, are more likely to alter the composition of microbiota and increase the serum concentration of TMAO (Figure 1) [110]. Therefore, the current generation may be at high risk of elevated TMAO and dysbiosis. A number of investigations have demonstrated that TMAO accelerates kidney injury. A high dietary intake of TMAO exacerbates tubular injury and promotes renal fibrosis, with an increase in phosphorylated Smad3 shown in a rodent CKD model [17]. Recent animal data also showed that HFD induces renal fibrosis, with a corresponding increase in circulating TMAO, which could be inhibited by the administration of 3,3-Dimethyl-1-butanol (DMB), a trimethylamine formation inhibitor [111]. Interestingly, DMB attenuated renal inflammation with a reduction of oxidative stress in mice fed with HFD [111]. Not only is TMAO a contributor to the progression of kidney injury, but it also amplifies inflammation by directly regulating the function of immune cells. In basic research, TMAO has been shown to aggravate the severity of graft-versus-host disease by promoting alloreactive T cell proliferation in a rodent model [112]. TMAO also promoted the migration of macrophages in a CD36-dependent pathway in vitro [113]. That is supported by the evidence that Apo-E KO mice injected with TMAO for eight weeks exhibited enhanced macrophage infiltration into the aortic root [114]. These data suggest that the direct effect of TMAO on the function of immune cells could be the missing link between gut microbiota dysbiosis and inflammation in CKD. A recent study identified that organic anion transporter-3 (OAT-3), a major transporter expressed on the basolateral membrane of PTECs, mediates the excretion of TMAO into the urine. Since flosemide, a loop diuretic often prescribed in the clinical setting, competitively binds to OAT-3, CKD patients treated with flosemide are at a high risk of TMAO accumulation [115]. Similarly, protein-bound uremic toxins including IS and PCS are also taken up through OAT-1 or OAT-3 and secreted by PTECs. Probenecid inhibits the function of OAT-1 and OAT-3 [116]; thus, frequent use of those medicines might cause the accumulation of TMAO or uremic toxins.

## 3. Possible Interplay between Gut Microbiota and Uremic Toxins in CKD

### 3.1. AGEs and Dysbiosis in CKD

In addition to the intracorporeal formation of AGEs, dietary intake is another main source of AGEs since modern foods contain relatively high amounts. Cooking temperature, duration, pH, and method are shown to influence the generation of new AGEs in foods [117,118]. Meats including beef and poultry and meat-derived products such as sausages and bacon processed at high, dry heats contain large amounts of AGEs. High-fat cheeses and spreads such as butter, cream cheese, and margarine are also among the products with high dietary AGEs [119]. Importantly, dietary intake of AGEs is positively correlated with AGE levels in serum [120]. Recent studies have demonstrated that not only serum AGEs but also dietary AGEs are likely to be deposited in the gastrointestinal tract tissue and lead to the disruption of the microbiota (Figure 1) [29]. Given that dysbiosis is related to CKD progression, excessive intake of AGEs is likely to advance the progression of CKD and its comorbidities such as nerve system disorder [121], bone mineral disorder [122], cardiovascular diseases [12], and sarcopenia [123], possibly via the induction of dysbiosis. Mastrocola et al. explored the impact of an AGEs-enriched diet on inflammation, as well as on the composition of gut microbiota in mice. In an AGEs-rich diet group, N**ε**-CML deposition and the upregulation of RAGE were observed in the ileum portion of the intestine and submandibular salivary glands, with an increase in systemic inflammation assessed by the levels of IL-1β, IL-17, and TNF-α [124]. Using fecal microbiota analysis, they found that high AGEs exposure in the intestine affected the gut microbiota population, characterized by the reduction of *Anaerostipes*, one of the butyrate-producing species [124]. Another investigation has demonstrated that a high-AGEs diet reduces the levels of saccharolytic bacteria such as *Ruminococcaceae* and *Alloprevotella*, which are related to SCFAs’ production. These findings suggest that intestinal AGEs exposure can reduce SCFA production, which affects the risk of the progression of kidney injury [125]. Further, high amounts of dietary AGEs exposure downregulate tight junction proteins in the epithelia, such as zonula occludens-1 and claudin-5 [125]. A recent study investigating intestinal permeability, assessed in vivo by the clearance of FITC-labelled dextran, clearly showed that the intake of high dietary AGEs disrupts the gut epithelial barrier, inducing a “leaky gut” in db/db mice, as shown with a type 2 diabetic mouse model [69]. Thus, an increase in the uptake of AGEs affects the composition of gut microbiota, leading to a “leaky gut” via the disruption of the tight junctions of intestinal epithelial cells, which may go on to affect the progression of CKD (Figure 1). The restriction of dietary AGEs, or removing AGEs pharmacologically in the gastrointestinal tract can, therefore, be a potential therapeutic intervention option to preserve gut microbiota ecology, and consequently, inhibit the progression of CKD.

Several strategies using anti-AGEs have been evaluated in recent years. A clinical study demonstrated that AST-120, known to adsorb uremic toxins in the gut leading to its fecal excretion, decreased the serum concentrations of both glycated AGEs and Nε-CML in patients undergoing hemodialysis [126]. A recent single-center, randomized, open-label crossover study has demonstrated that sevelamer carbonate, a non-absorbed phosphate-binding polymer, substantially reduced the concentration of serum AGEs, with a decrease in inflammation and oxidative stress markers in diabetic CKD patients [127,128]. On the other hand, a large proportion of dietary AGEs are degraded and metabolized by the colonic microflora [129]. *Lactobacilli*, a commercially available probiotic supplement, is capable of producing glyoxalase, the enzyme that degrades dietary AGEs [57]. Therapy targeting probiotics is thus promising for blocking the interaction between dysbiosis and enhanced AGEs accumulation in the CKD condition. Further studies using larger human cohorts are required to confirm this observation.

### 3.2. AGEs–RAGE Coordinates with TMAO to Progress CKD

Animal products such as red meat, eggs, and fish are major dietary sources of betaine and L-carnitine, precursors of TMAO. Cooking these products at high temperatures (grilling, broiling, and frying) leads huge amounts of AGEs-bound proteins to accumulate, leading to an increase in the concentration of TMAO as well as those of AGEs (Figure 1). Mitchell et al. demonstrated that high protein intake (1.6 g/kgBW/day) increases the circulating concentration of TMAO in the elderly [130]. A recent epidemiological investigation showed that the amount of protein intake correlates with serum N**ε**-CML levels (Table 1) [131]. Based on these findings, a low protein diet, recommended as per CKD guidelines in several countries, is expected to have a better outcome in reducing the accumulation of TMAO and AGEs in the body. Further, a few studies have shown that the population of *Prevotella copri (P. copri)*, a gram-negative bacteria producing TMA (Table 1) [15], prospers due to an excess intake of AGEs [28,132], suggesting that AGEs may be associated with TMAO production by affecting the microbial composition in the gut. Tahara et al. also demonstrated that the serum AGEs/soluble RAGE (sRAGE) ratio has a positive correlation with serum TMAO concentration [133]. Given that sRAGE eliminates circulating AGEs by functioning as a decoy receptor [134], this finding indicates that endothelial cells in individuals with high circulating TMAO are more likely to be exposed to circulating AGEs, leading to endothelial dysfunction. An oral L-carnitine supplement is frequently used in hemodialysis patients who show carnitine deficiency due to insufficient carnitine synthesis and loss through dialysis in over 30 countries globally [135]. In fact, a clinical study by Adachi et al. showed a decrease in serum-free carnitine level in hemodialysis (HD) patients compared to healthy individuals, which was inversely correlated with skin AGE levels [136]. Thus, the L-carnitine supplement is shown to reduce skin AGE levels [137] with a decrease in vascular injury markers in HD patients (Table 1) [138]. However, it should be noted that there is a risk that oral L-carnitine supplementation can increase serum TMAO levels. Since intravenous L-carnitine administration does not affect TMAO levels, the method of L-carnitine administration should be considered depending on the patient’s condition. Taken together, these findings point to the possible implication of a link between AGEs and TMAO. However, further studies are required to understand the relationship and identify a therapeutic target.

## 4. Conclusions

The accumulation of uremic toxins is one of the main characteristics of advanced CKD and is linked to the further progression of kidney injury. Clinical symptoms of uremic syndrome including nausea, vomiting, muscle cramp, tremor, and loss of consciousness are caused by the accumulation of uremic toxins consequent to a failure of renal excretion. In addition to the inability to eliminate uremic toxins, CKD-related gut dysbiosis is recently suggested to produce more uremic toxins. Further, intake of dietary toxins such as AGEs and TMA precursors affects the composition of gut microbiota producing more uremic toxins and disruption of the epithelial barrier, leading to leaking of endotoxins, such as LPS, into the body. Thus, systematic studies aimed at teasing the interplay between these factors will pave the way to better understanding the relationship between all of these players in the progression of CKD.

## Figures and Tables

**Table 1:** Previous studies indicating possible correlation between AGEs and TMAO.

Authors [Reference] (Number of Participants)	Intervention	Outcome
Mitchell et al. [130] (*n* = 20)	High protein diet (1.6 g/kgBW/day)	Serum TMAO↑
Brinkley et al. [131] (*n* = 2439)	High protein diet (≥1.2 g/kgBW/day)	Serum Nε-CML↑, Serum sRAGE↑
Yacoub et al. [28] (*n* = 20)	Restriction of dietary AGEs intake	Serum Nε-CML↓, Serum methylglyoxal-derivatives↓ *Prevotella copri*↓, *Bifidobacterium animalis*↓ *Alistipes indistinctus*↑, *Clostridium citroniae*↑
Adachi et al. [136] (*n* = 204)	Observational study in healthy subjects (*n* = 75) and HD patients (*n* = 129)	*Clostridium hathewayi*↑, *Ruminococcus gauvreauii*↑ Serum-free carnitine inversely correlates with skin AGEs
Tahara et al. [133]	Observational study in non-diabetic subjects	*Clostridium hathewayi*↑, *Ruminococcus gauvreauii*↑ Serum-free carnitine inversely correlates with skin AGEs
Fukami et al. [137] (*n* = 102, HD patients)	Oral L-carnitine supplementation (900 mg/d), six months	Skin AGEs↓ Serum-free carnitine inversely correlates with the decrease in skin AGEs
Fukami et al. [138] (*n* = 31, HD patients)	Oral L-carnitine supplementation (900 mg/d), six months	Vascular injury markers (sICAM-1, sVCAM-1)↓ Oxidative stress marker (MDA)↓ Serum AGE tends to be decreased TMA↑, TMAO↑

## Data Availability

No new data were created or analyzed in this study. Data sharing is not applicable to this article.

## Abbreviations

GFR	Glomerular filtration rate
CKD	Chronic kidney disease
ESRD	End-stage renal disease
AKI	Acute kidney injury
DKD	Diabetic kidney disease
SLE	Systemic lupus erythematosus
PTECs	Proximal tubular epithelial cells
PBMCs	Peripheral blood mononuclear cells
AGEs	Advanced glycation endproducts
RAGE	Receptor for advanced glycation endproducts
sRAGE	Soluble RAGE
N(ε)-CML	N(ε)-carboxymethyl lysine
TMAO	Trimethylamine N-oxide
TMA	Trimethylamine
IS	Indoxyl sulfate
PCS	p-cresol sulfate
LPS	Lipopolysaccharide
ROS	Reactive oxygen species
NF-ĸB	Nuclear factor-ĸB
TNF-α	Tumor necrosis factor-α
MCP-1	Monocyte chemoattractant protein-1
INF-1α	Interferon-1α
VEGF	Vascular endothelial growth factor
sICAM-1	Soluble forms of intracellular adhesion molecule-1
sVCAM-1	Soluble forms of vascular cell adhesion molecule-1
MDA	Malondialdehyde
TLR	Toll-like receptor
PAMPs	Pathogen-associated molecular patterns
DAMPs	Damage-associated molecular patterns
SCFAs	Short-chain fatty acids
DMB	3:3-Dimethyl-1-butanol
HFD	High-fat diet
OAT	Organic anion transporter
